# miR-let-7d attenuates EMT by targeting HMGA2 in silica-induced pulmonary fibrosis

**DOI:** 10.1039/c9ra01031a

**Published:** 2019-06-20

**Authors:** Xinghao Yu, Ruonan Zhai, Baoyong Hua, Lei Bao, Di Wang, Yiping Li, Wu Yao, Hui Fan, Changfu Hao

**Affiliations:** School of Public Health, Zhengzhou University No. 100 Science Avenue Zhengzhou Henan 450001 China yaowu@zzu.edu.cn haochangfu@126.com; The Third Affiliated Hospital of Zhengzhou University Zhengzhou Henan 450001 China fanhuifh@yeah.net

## Abstract

Silicosis is a serious occupational disease characterized by pulmonary chronic inflammation and progressive fibrosis. Epithelial-mesenchymal transition (EMT) of alveolar epithelial cells plays a vital role in silicosis. Recent studies discovered a variety of microRNAs (miRNAs) participating in fibrotic diseases. Here, we aimed to explore the function and mechanism of miRNA let-7d in the EMT process in silica-induced alveolar epithelial cells. To detect whether let-7d and its target HMGA2 were involved in silica-induced EMT, we established a silicosis mouse model and found that let-7d was down-regulated and HMGA2 was up-regulated in the silica-treated group. Then we applied an *in vitro* co-culture system to imitate the EMT process in A549 cells after silica treatment. The down-regulation of let-7d and up-regulation of HMGA2 were also observed *in vitro*. The knockdown of HMGA2 significantly inhibited the silica-induced EMT. Furthermore, we found that overexpression of let-7d could reduce the expression of HMGA2 and consequently inhibited the silica-induced EMT, whereas inhibition of let-7d increased the expression of HMGA2 and promoted the silica-induced EMT. In conclusion, let-7d negatively regulated silica-induced EMT and inhibited silica-induced pulmonary fibrosis, which might be partially realized by directly binding to HMGA2. Our data suggested that miRNA let-7d might have a potential protective effect in the fibrotic process and become a new therapeutic target for silicosis or other fibrotic diseases.

## Abstract 2

Silicosis is a serious occupational disease characterized by pulmonary chronic inflammation and progressive fibrosis.

## Graphical abstract

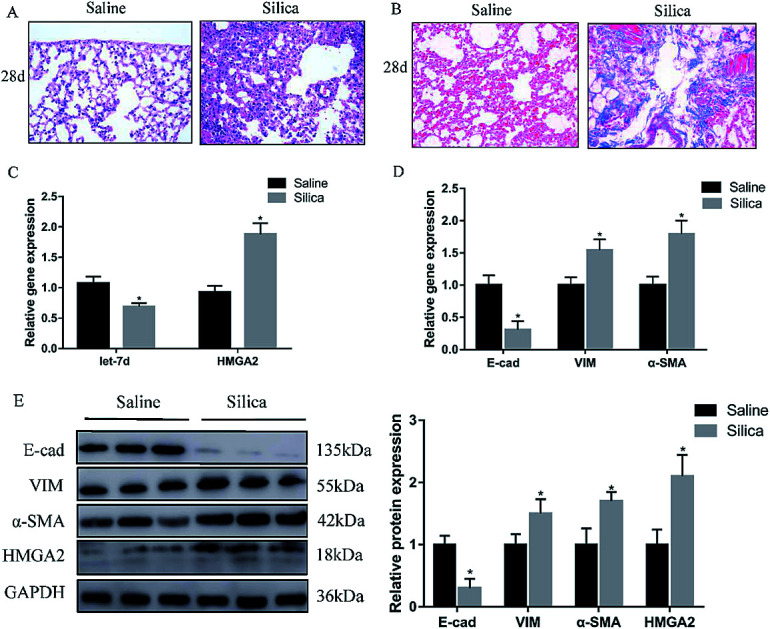

## 1 Introduction

Silicosis is an interstitial pulmonary fibrotic disease caused by inhalation of crystalline silica.^1^ There are approximately tens of millions of workers exposed to crystalline silica worldwide in both developing and developed countries.^2^ Occupational exposure is almost unavoidable in the industrialization in developing countries, although diverse measurements have been taken to prevent it.^3^ Long-term inhalation of crystalline silica can induce pulmonary chronic inflammation and progressive fibrosis, which is characterized by the production of collagen and excessive extracellular matrix deposition, leading to silicosis finally. So far, the molecular mechanism still hasn't been fully revealed, thus limiting the treatment of silicosis.

Emerging evidence suggests epithelial to mesenchymal transition (EMT) plays a critical role in the occurrence and progression of pulmonary fibrosis.^4,5^ During EMT, the epithelial cells lose their tight connection properties and acquire mesenchymal features, which is characterized by the decrease of E-cadherin (E-Cad) in epithelial cells and increased expression of mesenchymal proteins such as α-smooth muscle actin (αSMA).^6^ EMT is a complex biological process that is regulated by a variety of transcriptional regulators. High mobility group AT-hook 2 (HMGA2), as a key transcriptional regulator, plays a pivotal role in embryogenesis, EMT and tumor cell metastasis. Nowadays, more and more studies show that HMGA2 participated in organ fibrosis or tumor invasion process by regulating EMT.^7–9^

MicroRNAs (miRNAs) are small, non-coding RNAs normally consisting of 19–22 nucleotides. They could modulate gene expression both transcriptionally and posttranscriptionally and affect various biological processes.^10^ miRNAs dysregulation has also been reported to associate with various organs fibrotic disease.^8,11,12^ Previous studies showed that miRNAs participated in the process of EMT and pulmonary fibrosis. For instance, the decreased expression of miR-221 has been found in human idiopathic pulmonary fibrosis (IPF) tissues, and overexpression of miR-221 could inhibit bleomycin (BLM)-induced pulmonary fibrosis through the TGF-β1/Smad3 signaling pathway.^9^ The miR-200 family target ZEB1 and the miR-205 family target SIP1 to regulate EMT in tumor progression.^13^ MiR-448-5p inhibits TGF-β1-induced EMT and pulmonary fibrosis by targeting Six1.^14^ MiR-29c attenuates pulmonary fibrosis by regulating epithelial cell renewal and apoptosis.^15^

Let-7 was first identified in *Caenorhabditis elegans* and is highly conserved among *C. elegans*, *Drosophila*, and humans.^16^ Let-7 has been reported to significantly decrease during cancer progression and is closely related to tumor diagnosis, grading and prognosis.^17,18^ Moreover, recent studies have shown that let-7 participates in pulmonary and renal fibrosis by regulating EMT.^8,19^ Three miRNAs target databases (TargetScan, miRanda and PicTar) predicted HMGA2 to be a potential target of let-7.^20^ Although series of studies have confirmed the regulation relationship between let-7d and HMGA2 in IPF,^19^ renal fibrosis,^8^ ovarian cancer^21^ and aortic diseases,^22^ little is known about the relationship between let-7d and HMGA2 in silicosis.

In the present study, we established a mouse silicosis model to analyze the expression of let-7d and HMGA2. Then we used transwell to construct an *in vitro* co-culture model to detect the role of let-7d and HMGA2 in silica-induced EMT.

## 2 Materials and methods

### 2.1 Animals

Male Kunming mice (4–6 weeks of age) were purchased from the animal center of Henan province (Henan, China). The mice were housed under controlled temperature (22 ± 2 °C) and exposed to a 12 h light–dark cycle. All the cags, water and food were sterilized by autoclaving. The entire study was carried out in accordance with “Principles of Laboratory Animal Care and Use in Research” (State Council of China, 1988) and was approved by the Experiment Animal Ethics Committee of Zhengzhou University (Zhengzhou, China).

### 2.2 Generation of animal silicosis model

In this study, twelve mice were randomly divided into control and silica-treated groups (*n* = 6 in each group). Silica (SiO_2_ purity > 99%, average particle size 0.5–10 μm, Sigma Aldrich, Shanghai, China) was dried and then suspended in sterile saline at a concentration of 50 mg ml^−1^. The mice were anesthetized using pentobarbital sodium through intraperitoneal injection. Then the limbs and the head of mice were fixed with cotton thread. The muscles around the trachea were separated bluntly to exposure the trachea, and the mice were instilled with 50 mg kg^−1^ of silica in 0.05 ml sterile saline or 0.05 ml sterile saline intratracheally.

### 2.3 Euthanization procedure

After 28 days of installation, the mice were sacrificed with a sealed euthanasia device as previously described.^23^ In briefly, before the mice were placed into the device, we put a certain amount of carbon dioxide into the device, so the mice could enter anesthesia with reduced pain. Moreover, carbon dioxide was continuously passed for 2 to 3 minutes after the mice were euthanatized.

### 2.4 Histopathology measurement

The right lung tissues of the mouse were fixed in 10% neutral formalin and embedded in paraffin. Then 5 μm lung tissue section were stained with both hematoxylin and eosin (HE) for histological examination. In addition, Masson trichrome method was used to evaluate the fibrosis degree. Samples were examined under a microscope with a magnification of 100× by a pathologist in a blind fashion for assessment.

### 2.5 Cell lines and cell cultures

A549 cell lines and THP-1 cell lines were purchased from the Cell Bank of Type Culture Collection of the Chinese Academy of Sciences (Shanghai, China). Both of the cell lines were maintained in RPMI 1640 medium (Hyclone, South Logan, UT, USA) with 10% fetal bovine serum (FBS; Hyclone Laboratories, Logan, UT). Both medium were supplemented with 1% penicillin/streptomycin. Cells were maintained at 37 °C in a humidified incubator in an atmosphere of 95% air and 5% CO_2_.

### 2.6 CCK-8 assays

Approximately 5 × 10^4^ THP-1-induced macrophages were plated in 96-well plates. Then, the medium containing silica was diluted to the required concentrations in the cell culture medium and added to the cells. In this experiment, ten different concentrations, namely, 0, 25, 50, 75, 100, 125, 150, 175, 200, 225 μg ml^−1^, were chosen to treat the cells. After incubation for 48 h, the medium was discard and the cells were washed three times with PBS. Then, 10 μl of CCK-8 (Dojindo Laboratories, Kumamoto, JAPAN) in 100 μl of the medium was added to the cells in each well and incubated for 2 h at 37 °C. The absorbance at 450 nm was quantified a multimode plate reader. The experiments were performed in triplicate.

### 2.7 Co-culture of A549 cells and macrophages

The THP-1-induced macrophages and A549 cells were co-cultured using a six-well cell culture insert (Corning, NY, USA) with a 0.4 μm porous membrane to separate the upper and lower chambers. The THP-1 cells (5 × 10^5^ cells) were seeded into the upper chamber of the transwell, induced to macrophages by the addition of 100 ng ml^−1^ PMA (Sigma Chemical) for 48 h, washed three times with PBS, and incubated for another 24 h to eliminate the effect of PMA.^24^ The A549 cells (2 × 10^5^) were seeded in the six-well plates for 24 h to allow their adherence to the walls. The chamber with the THP-1-induced macrophages was placed directly on top of the six-well plates containing the attached A549 cells, and the THP-1-induced macrophages were treated with saline or silica. The resulting co-culture systems were cultured for another 48 h. After that, A549 cells were collected for qPCR or WB.

### 2.8 Enzyme-linked immunosorbent assay (ELISA)

To determine the amounts of TGF-β1 produced by the THP-1-induced macrophages, Elisa was performed according to the manufacturer's instructions. The THP-1-induced macrophages (5 × 10^5^ cells) were seeded into 6-well plates with or without silica. The cell culture supernatants were harvested, centrifuged, and placed at −80 °C. A human TGF-β1 Elisa Kit from Elabscience was used to detect the amount of TGF-β1. All assays were repeated three times.

### 2.9 Cell transfection

A549 cells were used in the transfection assays. SiRNA, control siRNA, miR-let-7d mimics, mimics control, miR-let-7d inhibitor, and inhibitor control was purchased from RiBoBio Co. (RiboBio, Guangzhou, China). Cells were transfected using Lipofectamine® 3000 (Invitrogen, Carlsbad, CA, USA) following the manufacturer's protocol. Briefly, 50 nM siRNA or NC (negative-control, NC), 50 nM miR-let-7d mimics or mimics NC, 100 nM miR-let-7d inhibitor or inhibitor NC was diluted in 125 μl serum-free RPMI-1640 medium, 5 μl Lipofectamine® 3000 was diluted in another 125 μl serum-free RPMI-1640 medium. Then the two medium were mixed and incubated for 5 minutes at room temperature. Transfection medium containing target siRNA or miRNA mimics/inhibitor was transferred to each well of the culture plates, after incubation at 37 °C for 6 h, the medium was replaced by complete medium and the cells were co-cultured 48 h as previously described. After that, the cells were collected to further analysis.

### 2.10 qRT-PCR

Total RNA was extracted from lungs and cells using RNAiso Plus (TaKaRa, Tokyo, Japan). The isolated total RNA was reverse transcribed using Mir-X miRNA First-Stand Synthesis Kit (TaKaRa, Tokyo, Japan) for microRNA let-7d and the PrimerScript™ RT Reagent Kit with gDNA Eraser (TaKaRa, Tokyo, Japan) for mRNA, according to manufacturer instructions. Relative expression was assessed by ABI7500 Fast Real-Time PCR System (Applied Biosystems, USA) with the TB Green Premix Ex II (TaKaRa, Tokyo, Japan). Relative expression was calculated using the 2^−ΔΔ*C*_T_^ method and was normalized to the expression of U6 or GAPDH. ALL qRT-PCR reactions were performed in triplicate. The sequences of primer pairs are described in Table 1.

**Table 1:** The primer sequences of genes for qRT-PCR^a^

Gene/microRNA	Forward primer	Reverse primer
Ms/Hsa miR-let-7d	CCGCGTGAGGTAGTAGGTTGTATAGTT	(TaKaRa, Tokyo, Japan)
Ms/Hsa U6	GGAACGATACAGAGAAGATTAGC	TGGAACGCTTCACGAATTTGCG
Hsa α-SMA	ATTGCCGACCGAATGCAGA	ATGGAGCCACCGATCCAGAC
Hsa E-cad	AGGATGACACCCGGGACAAC	TGCAGCTGGCTCAAGTCAAAG
Hsa HMGA2	AGCGCCTCAGAAGAGAGGAC	GAGCTGCTTTAGAGGGACTCTTGT
Hsa vimentin	CCTTGAACGCAAAGTGGAATC	GACATGCTGTTCCTGAATCTGAG
Hsa GAPDH	GCACCGTCAAGGCTGAGAAC	TGGTGAAGACGCCAGTGGA
Ms α-SMA	GCTCTGCCTCTAGCACACAA	ATTCCTGACCACTAGAGGGGG
Ms E-cad	CAGGTCTCCTCATGGCTTTGC	CTTCCGAAAAGAAGGCTGTCC
Ms HMGA2	CAGCTTGTCCCTCTGCATCT	ACCGAGGAGAGAGTGGAAGT
Ms vimentin	CCAGAGAGAGGAAGCCGAAA	CCACATCGATCTGGACATGG
Ms GAPDH	GTGAAGCAGGCATCTGAGGG	CGAAGGTGGAAGAGTGGGAGT

a Ms, mouse; Hsa, homo sapiens.

### 2.11 Western blotting

The proteins were extracted from lung and cells using RIPA buffer (Boster Biological Technology, Ltd, Wuhan, China) containing protease inhibitors (Boster Biological Technology). The BCA protein assay kit (Boster Biological Technology) was used to measure the total protein concentrations. Total protein was subjected to 8–15% SDS-PAGE polyacrylamide gel (Boster Biological Technology) and transferred to PVDF membranes (Boster Biological Technology). The PVDF membranes were blocked with 5% nonfat dry milk for 2 h at room temperature, then incubated the primary antibodies overnight at 4 °C. The used primary antibodies were as followed: anti-HMGA2 (1 : 1000; Cell Signaling Technology), anti-GAPDH (1 : 1000; Cell Signaling Technology), anti-α-SMA (1 : 1000; Cell Signaling Technology), anti-vimentin (1 : 1000; Proteintech), anti-E-cad (1 : 1000; Cell Signaling Technology). After incubating with the primary antibodies, the membranes were washed with TBST three times. Then the membranes were incubated with the secondary antibody, HRP-conjugated Affinipure Goat Anti-Rabbit IgG (1 : 5000; Proteintech) for 1 h at room temperature. The signals were detected using ECL detection reagent (Cwbiotech, China). The images were quantified by Image J software (Bethesda, MD, USA). When performing WB quantification, one of the control groups was set as the standard “1”, and the other groups were compared with it. All experiments were performed in triplicate.

### 2.12 Luciferase reporter assays

The HMGA2 wild-type (Wt) and mutant (Mut) 3′ UTR were conducted and cloned to the firefly luciferase-expressing vector psiCHECK (Promega). A549 cells were seeded in 24-well plates 24 h before transfection and cotransfected with the 0.5 μg HMGA2 Wt or Mut 3′ UTR report vector, and 50 nM let-7d mimics and NC using Lipofectamine® 3000. 48 h after transfection, luciferase activities were determined with the Dual-Luciferase Reporter System (Promega). Each transfection experiment was independently repeated three times.

### 2.13 Statistical analysis

SPSS 21.0 software was used for statistical analysis. All the data were presented as mean ± SD. The Student's *t*-test was used for comparisons of two independent groups and one-way analysis of variance (ANOVA) was used for more groups. Values of *p* < 0.05 were used to define as statistically significant.

## 3 Results

### 3.1 Let-7d was decreased and HMGA2 was increased in a mouse silicosis model

**Fig. 1:**
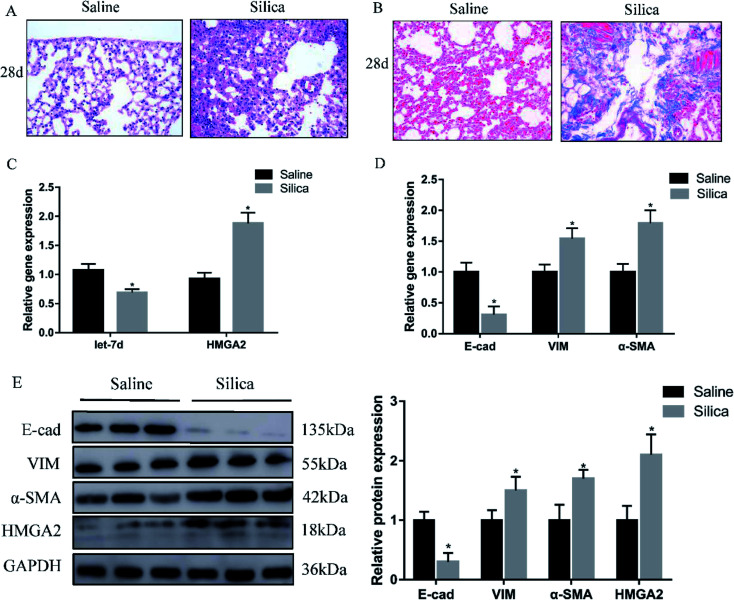
Let-7d is downregulated and HMGA2 is upregulated in silica-treated mice. HE (A) and Masson (B) staining of mouse lungs show interstitial fibrosis with collagen deposition 28 days after silica instillation. qPCR analysis of let-7d, HMGA2 and EMT-related genes mRNA levels in lung tissues (C and D). Western blot analysis of E-cad, VIM, α-SMA, HMGA2 and relative protein levels in silica-treated mice compared with the saline group (E). Means ± SEM; *n* = 6 mice in each group, *P < 0.05 *versus* saline group.

Since miRNA let-7d has been demonstrated as a direct regulator of HMGA2, we first analyzed their expression in a silica-induced pulmonary fibrosis mouse model. Mouse lungs were harvested 28 d after silica instillation. HE staining showed the appearance of fibrous tubercle, and the alveolar structure was destroyed in the silica-treated group (Fig. 1A). Masson's trichrome staining showed that large-scale dense collagen fiber was deposited in the silica-treated group but not in the saline-treated group (Fig. 1B). The expression of epithelial marker E-cad decreased significantly, whereas the mesenchymal markers α-SMA and vimentin showed increased expression both at the mRNA (Fig. 1D) and protein levels (Fig. 1E). qRT-PCR revealed the down-regulation of let-7d and the concomitant increase of HMGA2 in silica-treated mice (Fig. 1C and E), thus confirmed their potential relationship in silicosis *in vivo*. These results indicate that silica can induce pulmonary fibrosis and EMT, the decreased expression of let-7d and the increased expression of HMGA2 are involved in this process.

### 3.2 Silica induces increased expression of HMGA2 and EMT *in vitro*

**Fig. 2:**
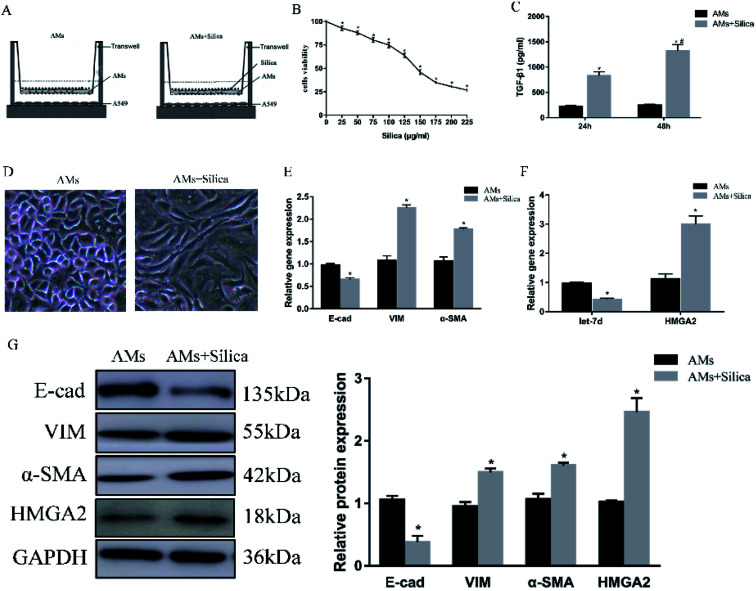
Effects of silica on the expression of EMT-related genes, let-7d, and HMGA2 in co-cultured A549 cells. Schematic design of transwell experiment (A). CCK-8 assays revealed the cell viability of silica on macrophages (B). THP-1-induced macrophages were treated with or without 150 μg ml^−1^ silica for 24 and 48 h. TGF-β1 secretion and synthesis levels were measured by Elisa (C). *P < 0.05 *versus* AMs group, ^#^*P* < 0.05 *versus* silica-treated 24 h group. The morphological changes in saline and silica treated A549 cells (D). qPCR analysis of EMT related genes (E) and let-7d, HMGA2 (F) in co-cultured A549 cells. Western blot analysis of proteins of EMT-related proteins and HMGA2 in A549 cells and quantification analysis was conducted and shown as a graph (G). Means ± SEM (*n* = 3), *P < 0.05 *versus* AMs group.

To further explore the mechanism in detail, we designed an *in vitro* co-culture system using transwell with THP-1-induced macrophages cultured in the upper well and A549 cells in the lower well (Fig. 2A). After 48 h exposure to silica, cell viability of THP-1-induced macrophages was measured by CCK-8 assay. Silica-stimulation resulted in decreased viability compared with controls (*P* < 0.05). Almost 50% of the cell died when silica concentration reached 150 μg ml^−1^ (Fig. 2B). Once the silica was consumed by macrophages, different types of cytokines were secreted, the main pro-fibrotic factor is TGF-β1. So we detected the levels of TGF-β1 in 150 μg ml^−1^ silica-treated macrophages by Elisa, the result showed that silica could promote the secretion of TGF-β1. The amount of TGF-β1 released by silica stimulation for 48 h was more than that of 24 h (Fig. 2C). Silica exposure induced morphological changes from a tight junction epithelial-like morphology to a fusiform mesenchymal-like morphology (Fig. 2D). This was consistent with the decreased expression of epithelial marker E-cad and the increased expression of mesenchymal markers α-SMA and vimentin both at the level of mRNA (Fig. 2E) and protein (Fig. 2G). Moreover, silica treatment caused a significant decrease in the expression of let-7d and increased transcription and translation of HMGA2 as observed in the mouse model (Fig. 2F), suggesting that let-7d may participate in the silica-induced EMT process in co-cultured A549 cells by regulating the expression of HMGA2.

### 3.3 siRNA targeting HMGA2 inhibits silica-induced EMT

**Fig. 3:**
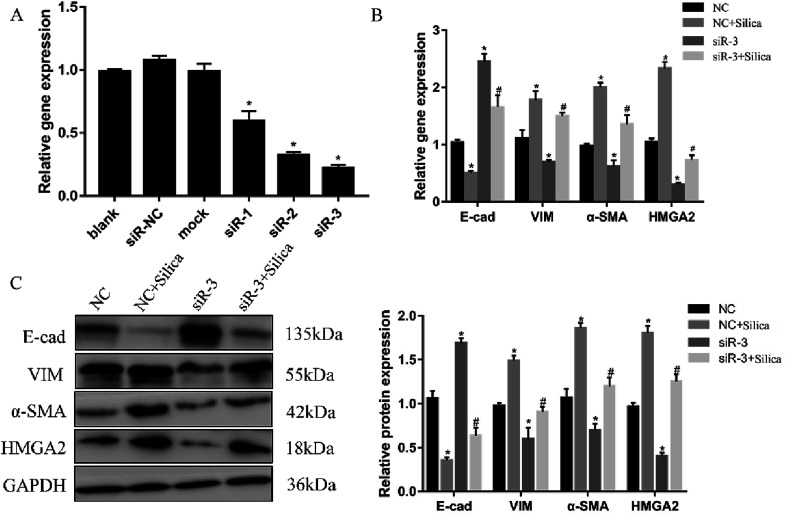
Knockdown of HMGA2 in the silica-treated A549 cells. Three siRNA targeting HMGA2 were transfected into A549 cells and the knockdown was measured by qRT-PCR (A). *P < 0.05 *versus* blank group. qRT-PCR analysis of EMT-related genes and HMGA2 mRNAs in A549 cells (B). Western blot analysis of protein of EMT-related genes and HMGA2 in A549 cells and the results of quantification analysis (C). Means ± SEM (*n* = 3), *P < 0.05 *versus* NC group, ^#^*P* < 0.05 *versus* NC + silica group.

Both *in vivo* and *in vitro* systems have exhibited the upregulated HMGA2 expression in silica-induced EMT, we thus continued to investigate whether HMGA2 was a vital transcription factor in this process by conducting siRNA-mediated knockdown of HMGA2 in A549 cells. All of the three siRNA could reduce the mRNA amount of HMGA2 (Fig. 3A). The siR-3 was selected for subsequent experiments because of its high inhibition efficiency. We performed qPCR and Western blot analysis to evaluate the levels of EMT markers and HMGA2 after silica treated. Comparing with the control group, knockdown of HMGA2 *via* transfection of siR-3 led to a significant decrease in the transcription and translation of HMGA2 as well as the mesenchymal markers vimentin and α-SMA, but enhanced the expression of epithelial marker E-cad (Fig. 3B and C). These result confirmed the direct regulation of HMGA2 as a vital transcription factor in silica-induced EMT in A549 cells.

### 3.4 HMGA2 is a direct target of let-7d

**Fig. 4:**
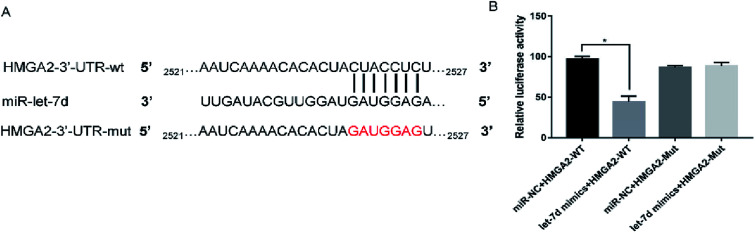
HMGA2 is a direct target of let-7d. Let-7d targeted the wild-type sequences or mutated sequences from the 3′-UTR of the HMGA2 gene (A). Effects of let-7d mimics on HMGA2 3′-UTR luciferase reporters in A549 cells. The luciferase activities were calculated as the ratio of firefly/Renilla activities and normalized to the miR-NC + HMGA2-WT group (B). *P < 0.05 *versus* the miR-NC + HMGA2-WT group.

**Fig. 5:**
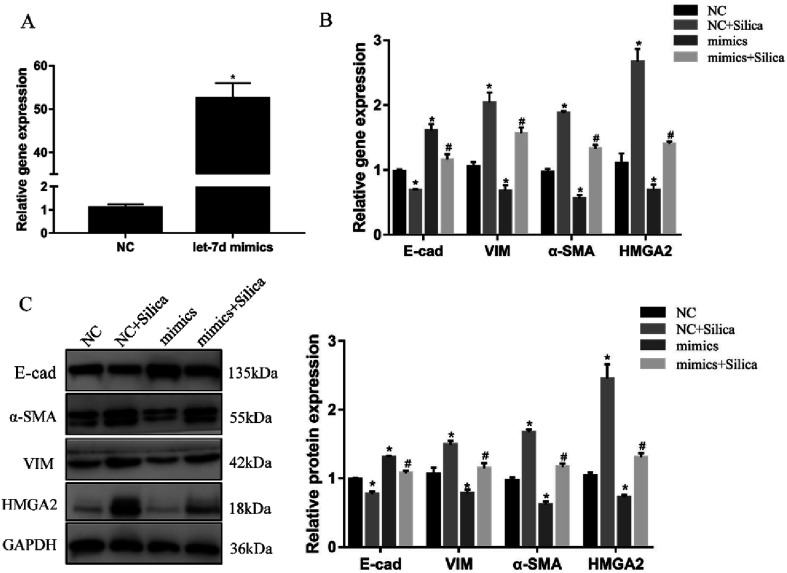
Overexpression of let-7d suppresses HMGA2 and EMT in silica-treated A549 cells. A549 cells were transiently transfected with 50 nM let-7d mimics, and the expression of let-7d was detected by qRT-PCR after 48 h (A). *P < 0.05 *versus* NC group. A549 cells were firstly transfected with 50 nM let-7d mimics 6 hours, and then co-cultured with or without silica for 48 h. Gene expression was measured by qRT-PCR for EMT-related genes and HMGA2 (B). The EMT-related genes and HMGA2 protein levels were measured by Western blot, and quantification analysis was conducted and shown as a graph (C). Means ± SEM (*n* = 3), *P < 0.05 *versus* NC group, ^#^*P* < 0.05 *versus* NC + silica group.

Let-7d was known to be an important regulator of HMGA2. To verify whether let-7d was capable of regulating HMGA2 by binding to the sites of it's 3′-UTR, A549 cells were transfected with let-7d mimics and a wild-type or mutant 3′-UTR vector (Fig. 4A) and performed luciferase reporter assay to determine whether HMGA2 was a direct target of let-7d. The results showed that the activity of the wild-type 3′-UTR reporter gene was inhibited but not the mutant report gene (Fig. 5B), indicating that let-7d can bind to the 3′-UTR of HMGA2.

### 3.5 Overexpression of let-7d reduces the expression of HMGA2 and attenuates EMT

We further investigated the effects of let-7d overexpression on HMGA2 and silica-induced EMT in A549 cells. Transfection of let-7d mimics could significantly increase the expression of let-7d (Fig. 5A) and reduce the expression of HMGA2 (Fig. 5B). In addition, it enhanced the expression of epithelial marker E-cad, while reduced the expression of mesenchymal markers both at the mRNA (Fig. 5B) and protein levels in A549 cells (Fig. 5C). These results demonstrated that overexpression of let-7d can reduce the expression of HMGA2 and attenuate the silica-induced EMT *in vitro*.

### 3.6 Inhibition of let-7d increase the expression of HMGA2 and promotes EMT

**Fig. 6:**
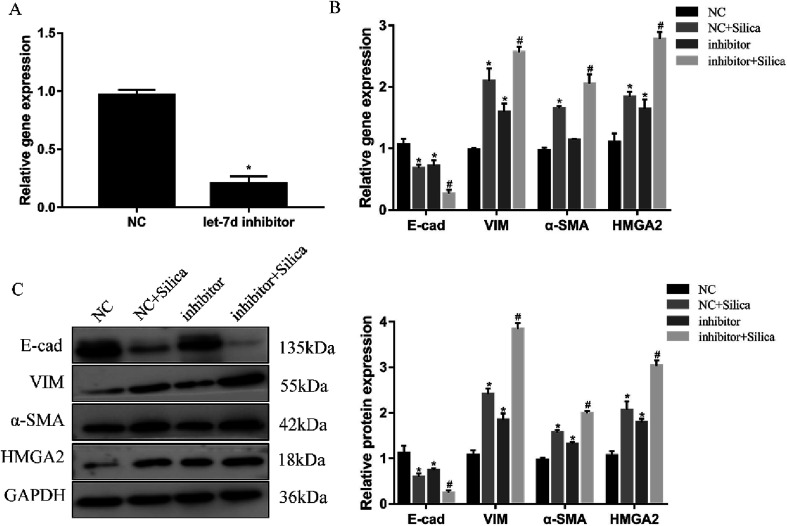
Effects of let-7d inhibitor on HMGA2 and silica-induced EMT in A549 cells. A549 cells were transiently transfected with 100 nM let-7d inhibitor, and the expression of let-7d was measured by qRT-PCR after 48 h (A). *P < 0.05 *versus* NC group. The EMT-related genes and HMGA2 were measured by qRT-PCR in A549 cells transfected with let-7d inhibitor (B). The EMT-related genes and HMGA2 protein levels were detected by Western blot, and quantification analysis was conducted and shown as a graph (C). Means ± SEM (*n* = 3), *P < 0.05 *versus* NC group, ^#^*P* < 0.05 *versus* NC + silica group.

Inhibition of let-7d in A549 cells by transfection with let-7d inhibitor was performed to further explore its functional significance in silica-induced EMT. Transfecting let-7d inhibitor significantly decreased the expression of let-7d (Fig. 6A). In addition, inhibition of let-7d increased the expression of HMGA2 and promoted the silica-induced EMT by up-regulating the mesenchymal markers while down-regulating the epithelial marker both at the mRNA and protein levels (Fig. 6B and C). These results indicated that inhibiting the expression of let-7d could aggravate EMT and promote fibrogenesis *in vitro*.

### 3.7 Let-7d modulates EMT by affecting the expression of HMGA2

**Fig. 7:**
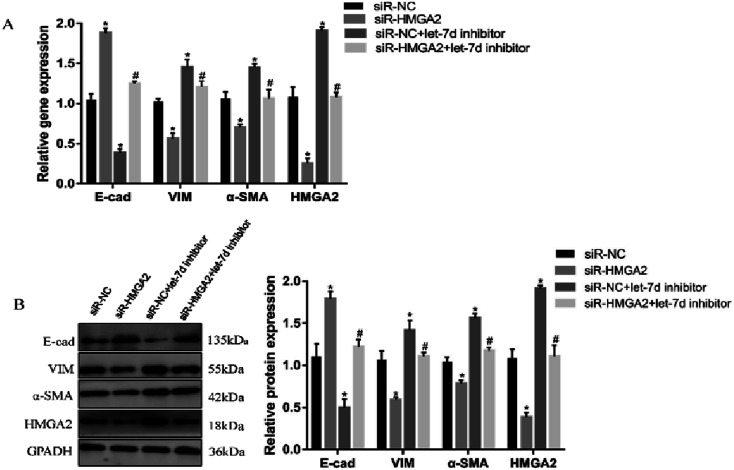
Let-7d modulates the EMT by regulating the expression of HMGA2. A549 cells were transfected with 50 nM siR-HMGA2 alone, 100 nM let-7d inhibitor alone, or both co-transfected. The EMT-related genes were detected by qRT-PCR (A). The protein levels was detected by Western blot and quantification analysis was conducted and shown as a graph (B). Means ± SEM (*n* = 3), *P < 0.05 *versus* siR-NC group, ^#^*P* < 0.05 *versus* siR-HMGA2 group.

To further verify if let-7d regulates the EMT of A549 by HMGA2, we transfected siR-HMGA2 alone, let-7d inhibitor, or both in A549 cells and then detected the expression of EMT-related genes (Fig. 7). Co-transfection of siR-HMGA2 with let-7d inhibitor reversed the EMT process due to the elevation of HMGA2 in A549. These data showed that HMGA2 could counteract the function of let-7d in the process of EMT.

## 4 Discussion

In this study, we first constructed a mouse silicosis model and demonstrated that EMT was involved in the process. The down-regulation of let-7d and the high-regulation of HMGA2 were also observed in silicosis. To further explore the role of let-7d and HMGA2 in silica-indued EMT, we constructed a co-culture model *in vitro* using THP-1-derived macrophages and A549 cells. Silica could induce the EMT and the same tendency of decreased expression of let-7d and increased expression of HMGA2 was confirmed *in vitro* system. Next, we silenced the HMGA2 gene in A549 cells using siRNA technique and observed the inhibition of EMT process, suggesting that HMGA2 plays an important role in silica-induced EMT. Considering the negative correlation between let-7d and HMGA2, overexpression and inhibition of let-7d were performed separately in the *in vitro* system. The data showed that up-regulation of microRNA let-7d lead to a down-regulation of HMGA2, which subsequently prevented silica-induced EMT, and vice verse. Thus, it is proved that microRNA let-7d participated in EMT during silica-induced fibrosis *via* its negative regulation of target gene HMGA2.

Chronic inhalation of silica is associated with the development of silicosis, which is characterized by pulmonary chronic inflammation and progressive fibrosis. Its specific mechanism is not completely clear. Emerging evidence suggests that multi-type cells and cytokines are involved in the pathological process of silicosis.^25–28^ Activated myofibroblasts are critical cells which can secrete collagen and lead to extensive pulmonary fibrosis.^29^ Therefore, understanding the source of myofibroblasts could contribute to the prevention and treatment of silicosis. Previous studies have shown that EMT is a vital source of myofibroblasts.^30–32^ Tanjore H. *et al.*^33^ found that in lung fibrosis, approximately 33% of myofibroblasts were transformed from epithelial cells through EMT. Therefore, further study on the mechanism of EMT is helpful to understand the pathogenesis of silicosis. Our study has demonstrated for the first time that specifically targets let-7d and HMGA2 can modulate the silica-induced EMT and attenuate fibrosis in A549 cells.

Once silica is inhaled into the lungs, the alveolar macrophages (AMs) are activated and begin to engulf silica. Then the activated AMs secrete multiple inflammatory factors and pro-fibrotic factors, such as IL-1β, TNF-α, IL-6, TGF-β, *etc.*^34^ In order to imitate this process, we used transwell to conduct a co-culture system in which the upper chamber was macrophages and the lower chamber was A549 cells. When the macrophages were stimulated with silica, they secreted various cytokines that could pass through the membrane of transwell to modulate A549. Our results showed after silica exposure, the A549 cells were detected to undergo EMT accompanied by low expression of let-7d and high expression of HMGA2. The results are in line with previous studies showing that TGF-β1 can employ HMGA2 to induce EMT in Idiopathic Pulmonary Fibrosis (IPF).^19^ HMGA2 is an important regulator in the EMT process. Tan EJ *et al.*^35^ found that in mammary epithelial cells, HMGA2 could bind to the promoter of Snail and induce Snail1 expression, E-cad repression, and the overall EMT. However, little studies have focused on the role of HMGA2 in the process of silica-induced EMT. In our study, we have found that silica stimulation leads to an increase in HMGA2 mRNA and protein levels. To further analyze the regulation of HMGA2 on EMT and silicosis, we used siRNA targeting HMGA2 to reduce the expression of HMGA2. Transfection of HMGA2 siRNA significantly decreased the expression of HMGA2 and it also reversed the silica-induced EMT, which suggests that HMGA2 may be an important regulator in silica-induced EMT. Recently, the first trial of using siRNA technology drug Onpattro was approved in the U.S. Food and Drug Administration (FDA), meaning that RNAi drugs have been applied for clinical treatment. Emerging evidence suggests that suppressing the abnormal expression of HMGA2 could reverse EMT in fibrosis disease,^8,9,36^ so using the RNA interference to silence the HMGA2 expression may be a novel therapeutic strategy.

Recent studies have found that several miRNAs have participated in pulmonary fibrosis.^9,12,37^ Let-7d is a member of the let-7 family which regulates cell proliferation, differentiation, and stem cell biology.^20^ Kusum *et al.*^19^ found that the let-7d was a key regulatory in preventing lung fibrosis. Matsuura *et al.*^38^ found that the rate of let-7d decline in chronic hepatitis C patients correlated with fibrosis progression. It is known that microRNA can modulate gene expression by interacting with target mRNAs *via* blocking translation or promoting degradation.^39^ Previous studies have suggested that HMGA2 is the target gene of let-7d.^8,21,40^ Our findings are consistent with their studies. This study has demonstrated for the first time that let-7d was sufficient to attenuate the effects of silicosis on pulmonary fibrosis through the repression of its target HMGA2 in A549 cells. However, there are some limitations to the present study: we simply confirmed that altering let-7d expression could affect silica-induced EMT *in vitro*, but whether the change of let-7d expression *in vivo* could cause the same changes remains to be verified.

In conclusion, we discovered the profound effects of HMGA2 on EMT induced by silica in A549 cells. Our study showed that let-7d could prevent silica-induced EMT by targeting HMGA2 expression. Given the pivotal role of microRNAs and their specific targets, further understanding of these effects will provide viable therapeutic avenues for the prevention and treatment of silicosis.

## Conflicts of interest

None of the authors has a financial relationship with a commercial entity that has an interest in the subject of this manuscript.

## Supplementary Material

## References

[cit1] Abdelaziz R. R., Elkashef W. F., Said E. (2016). Int. Immunopharmacol..

[cit2] Leung C. C., Yu I. T., Chen W. (2012). LANCET.

[cit3] Barmania S. (2016). Lancet Respir. Med..

[cit4] Guo J., Yang Z., Jia Q., Bo C., Shao H., Zhang Z. (2019). Toxicol. Lett..

[cit5] Guo R., Lv Y., Ouyang Y., Liu S., Li D. (2017). J. Cell. Biochem..

[cit6] Chen T., You Y., Jiang H., Wang Z. Z. (2017). J. Cell. Physiol..

[cit7] Hawsawi O., Henderson V., Burton L. J., Dougan J., Nagappan P., Odero-Marah V. (2018). Biochem. Biophys. Res. Commun..

[cit8] Wang Y., Le Y., Xue J. Y., Zheng Z. J., Xue Y. M. (2016). Biochem. Biophys. Res. Commun..

[cit9] Wang Y. C., Liu J. S., Tang H. K., Nie J., Zhu J. X., Wen L. L., Guo Q. L. (2016). Int. J. Mol. Med..

[cit10] Chen K., Rajewsky N. (2007). Nat. Rev. Genet..

[cit11] Hyun J., Wang S., Kim J., Rao K. M., Park S. Y., Chung I., Ha C. S., Kim S. W., Yun Y. H., Jung Y. (2016). Nat. Commun..

[cit12] Lei G. S., Kline H. L., Lee C. H., Wilkes D. S., Zhang C. (2016). Am. J. Pathol..

[cit13] Gregory P. A., Bert A. G., Paterson E. L., Barry S. C., Tsykin A., Farshid G., Vadas M. A., Khew-Goodall Y., Goodall G. J. (2008). Nat. Cell Biol..

[cit14] Yang Z. C., Qu Z. H., Yi M. J., Shan Y. C., Ran N., Xu L., Liu X. J. (2019). J. Cell. Physiol..

[cit15] Xie T., Liang J., Geng Y., Liu N., Kurkciyan A., Kulur V., Leng D., Deng N., Liu Z., Song J., Chen P., Noble P. W., Jiang D. (2017). Am. J. Respir. Cell Mol. Biol..

[cit16] Bartel D. P. (2004). Cell.

[cit17] Sun X., Liu J., Xu C., Tang S. C., Ren H. (2016). J. Cell. Mol. Med..

[cit18] Perdas E., Stawski R., Nowak D., Zubrzycka M. (2016). Int. J. Mol. Sci..

[cit19] Pandit K. V., Corcoran D., Yousef H., Yarlagadda M., Tzouvelekis A., Gibson K. F., Konishi K., Yousem S. A., Singh M., Handley D., Richards T., Selman M., Watkins S. C., Pardo A., Ben-Yehudah A., Bouros D., Eickelberg O., Ray P., Benos P. V., Kaminski N. (2010). Am. J. Respir. Crit. Care Med..

[cit20] Wu A., Wu K., Li J., Mo Y., Lin Y., Wang Y., Shen X., Li S., Li L., Yang Z. (2015). J. Transl. Med..

[cit21] Ye H., Chen J., Huang X., Guo A., Hao P. (2012). Nanfang Yike Daxue Xuebao.

[cit22] Belge G., Radtke A., Meyer A., Stegen I., Richardt D., Nimzyk R., Nigam V., Dendorfer A., Sievers H. H., Tiemann M., Buchwalow I., Bullerdiek J., Mohamed S. A. (2011). Histol. Histopathol..

[cit23] Bao L., Hao C., Liu S., Zhang L., Wang J., Wang D., Li Y., Yao W. (2018). RSC Adv..

[cit24] Dehai C., Bo P., Qiang T., Lihua S., Fang L., Shi J., Jingyan C., Yan Y., Guangbin W., Zhenjun Y. (2014). Immunol. Lett..

[cit25] Liu Y., Li Y., Xu Q., Yao W., Wu Q., Yuan J., Yan W., Xu T., Ji X., Ni C. (2018). Biochim. Biophys. Acta, Mol. Basis Dis..

[cit26] Zhang W., Zhang M., Wang Z., Cheng Y., Liu H., Zhou Z., Han B., Chen B., Yao H., Chao J. (2016). Toxicol. Appl. Pharmacol..

[cit27] Liu H., Fang S., Wang W., Cheng Y., Zhang Y., Liao H., Yao H., Chao J. (2016). Part. Fibre Toxicol..

[cit28] Li C., Lu Y., Du S., Li S., Zhang Y., Liu F., Chen Y., Weng D., Chen J. (2017). Theranostics.

[cit29] Liu Y., Xu H., Geng Y., Xu D., Zhang L., Yang Y., Wei Z., Zhang B., Li S., Gao X., Wang R., Zhang X., Brann D., Yang F. (2017). Respir. Res..

[cit30] Grande M. T., Sanchez-Laorden B., Lopez-Blau C., De Frutos C. A., Boutet A., Arevalo M., Rowe R. G., Weiss S. J., Lopez-Novoa J. M., Nieto M. A. (2015). Nat. Med..

[cit31] Deng H., Xu H., Zhang X., Sun Y., Wang R., Brann D., Yang F. (2016). Toxicol. Appl. Pharmacol..

[cit32] Shu D. Y., Lovicu F. J. (2017). Prog. Retinal Eye Res..

[cit33] Tanjore H., Cheng D. S., Degryse A. L., Zoz D. F., Abdolrasulnia R., Lawson W. E., Blackwell T. S. (2011). J. Biol. Chem..

[cit34] Kawasaki H. (2015). Inhalation Toxicol..

[cit35] Thuault S., Tan E. J., Peinado H., Cano A., Heldin C. H., Moustakas A. (2008). J. Biol. Chem..

[cit36] Liang H., Gu Y., Li T., Zhang Y., Huangfu L., Hu M., Zhao D., Chen Y., Liu S., Dong Y., Li X., Lu Y., Yang B., Shan H. (2014). Cell Death Dis..

[cit37] Su B., Zhao W., Shi B., Zhang Z., Yu X., Xie F., Guo Z., Zhang X., Liu J., Shen Q., Wang J., Li X., Zhang Z., Zhou L. (2014). Mol. Cancer.

[cit38] Matsuura K., De Giorgi V., Schechterly C., Wang R. Y., Farci P., Tanaka Y., Alter H. J. (2016). Hepatology.

[cit39] Chen X., Ba Y., Ma L., Cai X., Yin Y., Wang K., Guo J., Zhang Y., Chen J., Guo X., Li Q., Li X., Wang W., Zhang Y., Wang J., Jiang X., Xiang Y., Xu C., Zheng P., Zhang J., Li R., Zhang H., Shang X., Gong T., Ning G., Wang J., Zen K., Zhang J., Zhang C. Y. (2008). Cell Res..

[cit40] Lee S., Jung J. W., Park S. B., Roh K., Lee S. Y., Kim J. H., Kang S. K., Kang K. S. (2011). Cell. Mol. Life Sci..

